# Public support for healthy supermarket initiatives focused on product placement: a multi-country cross-sectional analysis of the 2018 International Food Policy Study

**DOI:** 10.1186/s12966-021-01149-0

**Published:** 2021-06-14

**Authors:** Clara Gómez-Donoso, Gary Sacks, Lana Vanderlee, David Hammond, Christine M. White, Claudia Nieto, Maira Bes-Rastrollo, Adrian J. Cameron

**Affiliations:** 1 Department of Preventive Medicine and Public Health, School of Medicine, University of Navarra, Pamplona, Spain; 2 Biomedical Research Centre Network on Physiopathology of Obesity and Nutrition (CIBERobn), Institute of Health Carlos III, Madrid, Spain; 3 Global Obesity Centre (GLOBE), Institute for Health Transformation, Deakin University, 221 Burwood Highway, Burwood VIC, Geelong, 3125 Australia; 4 Centre Nutrition, Santé et Société (NUTRISS), Institut sur la nutrition et les aliments fonctionnels, École de nutrition, Université Laval, Québec, Canada; 5 School of Public Health and Health Systems, University of Waterloo, Waterloo, Canada; 6 Centro de Investigación en Nutrición y Salud, Instituto Nacional de Salud Pública, Cuernavaca, Mexico

**Keywords:** Supermarket, Retail, Food environment, Attitudes, Diet, Food policy

## Abstract

**Background:**

Food retail environments have an influential role in shaping purchasing behavior and could contribute to improving dietary patterns at a population level. However, little is known about the level of public support for different types of initiatives to encourage healthy food choices in supermarkets, and whether this varies across countries or context. The current study aimed to explore the level of support for three potential supermarket initiatives focused on product placement across five countries, and factors that may influence this support.

**Methods:**

A total of 22,264 adults from Australia, Canada, Mexico, the United Kingdom and the United States (US) provided information on support for three supermarket initiatives related to product placement (targeting product positioning: ‘checkouts with only healthy products’, ‘fewer end-of-aisle displays containing unhealthy foods or soft drinks’ or availability: ‘more shelf space for fresh and healthier foods’) as part of the online 2018 International Food Policy Study. The proportion of respondents that supported each initiative was assessed across countries, and multivariable logistic regression analyses were conducted to evaluate the influence of sociodemographic factors on support.

**Results:**

The initiative that received the highest support was ‘more shelf space for fresh and healthier foods’: 72.0% [95% CI 71.3–72.7], whereas ‘checkouts with only healthy products’ received the lowest support: 48.6% [95% CI 47.8–49.4]. The level of support differed between countries (*p* < 0.001 for all initiatives), with the US generally showing the lowest support and Mexico the highest. Noteworthy, in the overall sample, there was not much opposition to any of the initiatives (2.5–14.2%), whereas there was a large proportion of neutral responses (25.5–37.2%). Respondents who were older, female, highly educated, and those who reported having more nutrition knowledge tended to be more supportive, with several differences between countries and initiatives.

**Conclusions:**

Most people in the assessed five countries showed a generally high level of support for three placement initiatives in supermarkets to encourage healthy food choices. Support varied by type of initiative (i.e., product positioning or availability) and was influenced by several factors related to country context and sociodemographic characteristics. This evidence could prompt and guide retailers and policy makers to take stronger action to promote healthy food choices in stores.

**Supplementary Information:**

The online version contains supplementary material available at 10.1186/s12966-021-01149-0.

## Introduction

Unhealthy diets represent one of the leading preventable risk factors for chronic diseases worldwide [1]. Many food retail environments currently encourage and promote unhealthy food choices, likely contributing to the increasing epidemic of obesity and diet-related chronic diseases [2–4]. Globally, large supermarket chains have become dominant players in the food retail industry, replacing traditional food markets and small specialized stores [5]. Marketing practices within supermarkets such as product displays, placement and promotions strongly influence food accessibility, availability, affordability and desirability, which in turn play an important role in shaping food preferences and purchasing behavior [6–11]. Multiple studies across a range of countries have shown how supermarket environments currently favor the promotion of less healthy food despite recommendations that these foods should only be consumed occasionally and in small amounts [12–19]. Even more worrying is that public health strategies such as front-of-pack nutrition labelling could be undermined by current retail food environments predominantly driving consumption of unhealthy foods. Given that supermarkets are the setting for more than 50% of all food purchased globally [20], and that more than half of consumers’ grocery store purchasing decisions are unplanned [21, 22], in-store environmental initiatives that promote healthy food choices have significant potential to improve dietary-related behaviors and lower the risk of disease at the population level [23–25].

Despite the influence of food environments on purchasing intentions and behaviors, policy actions to address diet-related chronic diseases have often focused on the individual level [26–28]. The limited implementation of policies to create healthier food environments may be due to lack of popular support for fiscal and regulatory interventions, which are sometimes deemed intrusive to individuals’ freedom of choice [29–34]. However, there is evidence showing high public support for nudges [35–38], which apply behavioral science approaches to make small, usually unnoticed, environmental changes that steer people in particular directions. Changes to food retail environments that impact the way choices are presented to consumers (i.e., choice architecture) are well-recognized examples of nudges that target non-deliberative decisions [39]. Although most nudges in modern supermarkets currently promote purchasing of less healthy products [14–19], they could equally promote the purchasing of healthier foods. Systematic reviews have indeed shown that healthy food retail interventions targeting the in-store supermarket environment have led to improvements in the healthiness of consumer purchases [8, 40]. These interventions have mainly focused on changes regarding product placement, such as altering the position or availability of certain products. Examples include increasing the range or number of healthy options or avoiding placement of less healthy options in prominent locations, like checkout lanes, end-of-aisle or island bin displays.

Previous studies in Europe, the United Kingdom (UK) and the United States (US) have demonstrated widespread customer support for health-promoting supermarket nudges related to product positioning (e.g., changing the shelf location of sugar-sweetened beverages, placing most healthy foods in a prominent location, and requiring sweet-free checkouts) [36–38]. Nevertheless, few studies have examined the public support for product placement supermarket interventions focused on both positioning and availability across different countries. The level of public support for particular initiatives is recognized as playing a pivotal role in determining the extent to which evidence is implemented into policy [41, 42]. Moreover, increased understanding of the level of public support for various initiatives in different contexts and the opportunities to influence support could guide food retailers’ efforts to shape food choices. Accordingly, this study aimed to evaluate the level of public support for several product placement supermarket initiatives that were selected based on policy relevance, feasibility for retailers or their likely public health impact, and the sociodemographic factors associated with support within and between countries.

## Methods

### Study design and participants

This study used data from the 2018 International Food Policy Study (IFPS), an annual, cross-sectional study conducted in Australia, Canada, Mexico, the UK, and the US. The IFPS was designed to evaluate the impact of public health nutrition interventions on dietary patterns and policy-relevant behaviors across countries that are introducing novel regulations in the area of food policy (e.g., food retail interventions, price/taxation, food packaging and labelling, food marketing and others). A self-administered web-based survey was completed in November–December 2018 by adults aged 18+ in each of the five countries, and collected information on sociodemographic characteristics, diet and food policy-related attitudes, behaviors and knowledge. Participants were recruited through the Nielsen Consumer Insights Global Panel and their partners’ panels (https://www.nielsen.com/us/en/about-us/panels/) using non-probability sampling methods based on quota requirements for age and sex to facilitate recruitment of a diverse sample that approximated the corresponding proportions in the general population of each country. Eligible panelists (i.e., individuals aged 18+ years living within the target countries) were invited by email to complete the survey. After screening for eligibility and quota requirements, all potential respondents were provided with information about the study and were asked to provide consent before participating. A total of 439,821 invitations were sent to panelists; of which 7.7% accessed the survey link and 6.5% completed the 2018 IFPS survey (*n* = 28,684). Respondents provided consent before completing the survey and were incentivized in accordance with their panel’s existing reward structure (e.g., points-based or monetary rewards, or chances to win prizes). The study was reviewed by and received ethics clearance through a University of Waterloo Research Ethics Committee (Office of Research Ethics #30829) prior to data collection. A full description of the study methods, including annual surveys conducted in each of the five countries, is available elsewhere [43].

### Measures

#### Support for supermarket initiatives focused on product placement

The current study analyzed survey questions on respondents support for three supermarket initiatives pertaining to product placement: 1) Checkouts with only healthy products (e.g., no soft drinks, chocolate, confectionery) (‘checkouts’), 2) Fewer end-of-aisle displays containing unhealthy foods or soft drinks (‘end-of-aisle’), and 3) More shelf space for fresh and healthier foods such as fruits and vegetables (‘shelf space’). These initiatives were classified using the TIPPME framework (Typology of Interventions in Proximal Physical Micro-Environments) [44], which was developed to reliably classify and describe nudging interventions based on altering small aspects of the environment to change health-related behavior at the population level. Accordingly, the initiatives were classified as product positioning interventions (initiatives limiting or banning unhealthy food from highly visible places: ‘end-of-aisle’ and ‘checkouts’) or availability interventions (initiatives increasing the range or number of healthy food: ‘shelf space’). Those included were mainly selected based on evidence on the effect of nudges on purchasing behavior (‘end-of-aisle’) [7, 45] and evidence that supermarket retailers can successfully implement the nudge (‘checkouts’ and ‘shelf space’) [46–49].

To reduce survey length and minimize respondent burden, respondents were shown a randomly selected subset of two of the three supermarket measures. Policy support was measured by asking respondents ‘Would you support or oppose the following practices in supermarkets...’ and completing the question with each of the actions stated above, shown in a randomized order. Respondents could select either ‘support’, ‘neutral’, ‘oppose’, ‘don’t know’ or ‘refuse to answer’. These responses were re-categorized into a binary variable for analysis (support/other), where responses of ‘support’ were categorized as ‘support’, and responses of ‘neutral’ and ‘oppose’ were categorized as ‘other’. Responses of ‘don’t know’ and ‘refuse to answer’ were excluded (*n* = 560). There were no missing data among respondents.

#### Sociodemographic variables

Self-reported sociodemographic variables collected in the survey included age, sex, ethnicity, education, and body mass index (BMI). For the current study, age was categorized into four age groups that were also used to facilitate recruitment of a diverse sample: 18–29, 30–44, 45–64, and 65+ years. Ethnicity was categorized as ‘majority’ (if respondents identified as white “only” in Canada, UK and USA, English speaking in Australia or non-indigenous in Mexico), ‘minority’ (other valid responses), or ‘not stated’. Education level was categorized as ‘low’ (i.e., secondary school completion or lower), ‘medium’ (i.e., some post-secondary qualifications), or ‘high’ (i.e., university degree or higher) according to country-specific criteria related to the highest level of formal education attained, or ‘not stated’. Further details about country-specific criteria and question wording can be found at http://foodpolicystudy.com/methods. Self-reported height and weight data were used to calculate BMI, which was categorized as ‘underweight’ (< 18.5 kg/m^2^), ‘normal weight’ (18.5–24.9 kg/m^2^), ‘overweight’ (25.0–29.9 kg/m^2^), ‘obesity’ (≥30.0 kg/m^2^), or ‘missing/not stated’. Self-reported nutrition knowledge was measured by asking ‘How would you rate your nutrition knowledge?’. Responses of ‘not at all knowledgeable’ and ‘a little knowledgeable’ were categorized as ‘none/low’, responses of ‘somewhat knowledgeable’ were categorized as ‘moderate’, and responses of ‘very knowledgeable’ and ‘extremely knowledgeable’ were categorized as ‘high’. For education, ethnicity, BMI and nutrition knowledge, responses of ‘don’t know’ and ‘refuse to answer’ were re-categorized as ‘not stated’. Participants in this category, as well as missing data (except for BMI), were excluded from the logistic regression analyses (*n* = 376) but included when reporting the proportion of respondents that support supermarket initiatives and descriptive characteristics of study participants.

#### Dietary variables

Respondents’ dietary behavior was assessed through the following self-reported measures: sugar-sweetened beverage (SSB) and fruits and vegetables (FV) consumption.

The Beverage Frequency Questionnaire (BFQ), a 7-day food record that assesses consumption for 24 types of drinks [50], was used to derive weekly SSB consumption. For each beverage category, respondents reported the number of drinks they had consumed over the past week and the usual portion size, using examples of beverages and category-specific images of beverage containers to prompt recognition. The BFQ was adapted for each country to provide product examples and typical beverage container sizes commonly sold in each market. Total volume for each beverage category of interest was calculated by multiplying the number of drinks consumed in the previous 7 days by the usual serving size selected for that category. Total SSB consumption included intake of regular soda, sweetened fruit drinks, flavored waters, sports drinks, energy drinks, flavored milk, specialty coffees like mochas or frappucinos, sweetened smoothies, protein shakes and drinkable yogurt. The total SSB consumption variable was categorized into three groups: ‘none’, ‘low’ (i.e., below the mean) and ‘high’ (i.e., above the mean) according to the weighted mean of weekly SSB consumption amongst consumers in the analytical sample (1888 mL).

As part of an assessment of general health status, respondents were asked the number of times they consumed fruit and vegetables (excluding non-100% fruit-juice and fried potatoes) per day, week or month during the past 30 days. This information was used to compute a daily FV consumption variable where, based on global recommendations on healthy diet [51], intake lower than 3 servings/day was categorized as ‘low’, intake between 3 and less than 5 as ‘moderate’ and intake equal to or higher than 5 as ‘high’.

### Statistical analyses

Data were weighted with post-stratification sample weights constructed using a raking algorithm with country-specific population estimates from census data based on age group, sex, region, ethnicity (except in Canada) and education (except in Mexico). A detailed explanation of survey weights can be found at http://foodpolicystudy.com/methods (International Food Policy Study: Technical Report 2018). These sample weights were used throughout the analysis in order to minimize the influence of differential non-response and selection bias on the representativeness of findings.

Descriptive statistics were used to summarize the sociodemographic characteristics of the sample. The proportion of ‘support’, ‘neutral’ and ‘oppose’ responses regarding the three supermarket interventions was determined overall (i.e., as a total sample) and by country. The percentage of overall supermarket action support using the binary support variable was also assessed for each country.

Multivariable logistic regression models were fitted using binary support for each supermarket initiative among respondents to explore associations between sociodemographic variables and support. Explanatory variables introduced in models included sex, age, education, BMI classification and self-reported nutrition knowledge. These were selected a priori to be included as covariates in the logistic regression models based on existing literature [32, 33]. Dietary behaviors, including SSB and FV consumption, were assessed as covariates in supplementary analyses. Adjusted OR (95% CI) of support for each supermarket initiative are presented adjusted for all other variables. This analysis was completed among the total sample and among each country individually. Two-way interactions between country and each of the covariates were assessed by including each interaction in the model and performing a contrast analysis.

Statistical significance was set at the conventional 0.05 level. Analyses were performed using Stata version 14.0 (StataCorp, College Station, TX).

## Results

### Sample characteristics

A total of 28,684 respondents completed the 2018 IFPS survey. Respondents were excluded for the following reasons: region was missing, ineligible or had an inadequate sample size (i.e., respondents from the 3 Canadian territories); invalid response to a data quality question; survey completion time under 15 min; and/or invalid responses to at least three of 16 open-ended measures (*n* = 5860). The analytic sample included 22,824 respondents. A sub-sample of 22,264 respondents (Australia: *n* = 4004; Canada: *n* = 4288; Mexico: *n* = 4082; United Kingdom: *n* = 5367; United States: *n* = 4523) were included in the current analysis after excluding those responding ‘don’t know’ or ‘refuse to answer’ regarding support for supermarket initiatives.

Weighted sample characteristics for each country are described in Table 1. The weighted mean age (± SD) among all participants was 46 (± 20) years, and the proportions of male and female respondents were approximately equal across the five countries. Among the overall sample, the majority of respondents reported having low education level and some nutrition knowledge, and were classified as having self-reported BMI between 18.5 and 24.9. As expected, with each participant randomly asked about their support for only two of the three supermarket initiatives in an attempt to restrict overall survey length, almost no variation was seen in unweighted sample characteristics for the subsets of respondents that answered each question (see Additional file 1).

**Table 1:** Weighted sociodemographic characteristics of participants in five countries (expressed as n (%), unless otherwise stated) from the International Food Policy Study 2018 (*n* = 22,264)

	Overall *n* = 22,264	Australia *n* = 4004	Canada *n* = 4288	United Kingdom *n* = 5367	United States *n* = 4523	Mexico *n* = 4082
Sex						
Male	10,909 (48.7)	1954 (48.8)	2123 (49.5)	2614 (48.7)	2203 (48.7)	1947 (47.7)
Female	11,355 (51.3)	2050 (51.2)	2165 (50.5)	2753 (51.3)	2320 (51.3)	2135 (52.3)
Age, mean (SD)	46.0 (20.1)	46.5 (19.6)	48.2 (21.9)	48.1 (20.4)	46.9 (20.3)	39.3 (18.3)
Age group						
18–29 years old	4965 (22.3)	877 (21.9)	853 (19.9)	1041 (19.4)	954 (21.1)	1233 (30.2)
30–44 years old	5878 (26.4)	1057 (26.4)	1055 (24.6)	1310 (24.4)	1140 (25.2)	1318 (32.3)
45–59 years old	5788 (26.0)	981 (24.5)	1111 (25.9)	1406 (26.2)	1149 (25.4)	1147 (28.1)
60+ years old	5633 (25.3)	1089 (27.2)	1269 (29.6)	1610 (30.0)	1280 (28.3)	384 (9.4)
Ethnicity						
Majority	17,611 (79.1)	3015 (75.3)	3293 (76.8)	4728 (88.1)	3406 (75.3)	3159 (77.4)
Minority	4386 (19.7)	977 (24.4)	858 (20.0)	596 (11.1)	1094 (24.2)	861 (21.1)
Not stated	267 (1.2)	12 (0.3)	137 (3.2)	43 (0.8)	23 (0.5)	62 (1.5)
Education						
Low	9484 (42.6)	1666 (41.6)	1809 (42.2)	2576 (48.0)	2632 (58.2)	812 (19.9)
Medium	4943 (22.2)	1297 (32.4)	1424 (33.2)	1229 (22.9)	448 (9.9)	535 (13.1)
High	7770 (34.9)	1033 (25.8)	1038 (24.2)	1530 (28.5)	1438 (31.8)	2731 (66.9)
Not stated	67 (0.3)	8 (0.2)	17 (0.4)	32 (0.6)	5 (0.1)	4 (0.1)
BMI						
< 18.5	668 (3.0)	124 (3.1)	142 (3.3)	161 (3.0)	158 (3.5)	86 (2.1)
18.5–24.9	7748 (34.8)	1441 (36.0)	1432 (33.4)	1852 (34.5)	1375 (30.4)	1633 (40.0)
25–30	6167 (27.7)	1053 (26.3)	1226 (28.6)	1428 (26.6)	1257 (27.8)	1208 (29.6)
≥ 30	4609 (20.7)	833 (20.8)	1029 (24.0)	896 (16.7)	1226 (27.1)	633 (15.5)
Missing/not stated	3072 (13.8)	553 (13.8)	459 (10.7)	1030 (19.2)	507 (11.2)	522 (12.8)
Nutrition knowledge						
None/low	8416 (37.8)	1457 (36.4)	1454 (33.9)	2581 (48.1)	1569 (34.7)	1359 (33.3)
Moderate	9462 (42.5)	1654 (41.3)	1908 (44.5)	1911 (35.6)	1841 (40.7)	2147 (52.6)
High	4275 (19.2)	873 (21.8)	892 (20.8)	848 (15.8)	1090 (24.1)	567 (13.9)
Not stated	111 (0.5)	20 (0.5)	34 (0.8)	27 (0.5)	23 (0.5)	9 (0.2)

### Support for supermarket initiatives focused on product placement

**Fig. 1:**
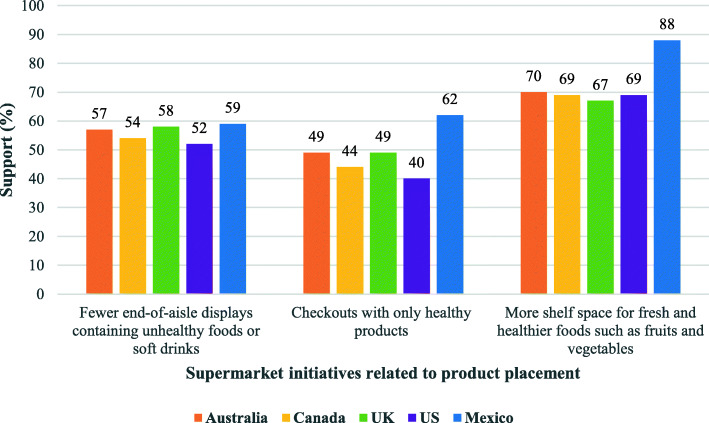
Weighted proportion of respondents that support supermarket initiatives related to product placement by country. International Food Policy Study 2018 (*n* = 22,264)

The proportion of respondents that supported each supermarket initiative by country is shown in Fig. 1, and is reported in more detail in Additional file 2 (i.e., proportion of neutral and opposition responses also shown). The level of support was relatively high for all initiatives, ranging between 48.6–72.0% across the total sample. The most supported initiative overall and across different countries was ‘more shelf space for fresh and healthier foods’, and the least supported initiative was ‘checkouts with only healthy products’. Consistent with the support trend, the initiative with the highest level of opposition overall was ‘checkouts with only healthy products’ and the initiative with the lowest opposition was ‘more shelf space for fresh and healthier foods’. However, in general, respondents across the total sample did not show much opposition to any of the initiatives (2.5–14.2%), and there was a large proportion of neutral responses to most initiatives (25.5–37.2%). For instance, while the lowest support across all countries was 48.6% for checkouts with only healthy products, only 14.2% opposed to this initiative (see Additional file 2).

The level of support differed significantly between countries (*p* < 0.001). Support for all initiatives was generally lowest in the US and highest in Mexico, although between-country differences varied across initiatives. In accordance with this trend, the level of opposition was highest in the US and lowest in Mexico.

### Sociodemographic characteristics and support for supermarket initiatives

Results of the multivariable logistic regression model fitted to explore associations between support and sociodemographic variables are shown in Table 2. Overall, respondents who were older, female, more highly educated and had greater self-reported nutrition knowledge tended to be more supportive. These findings remained substantially unchanged when a sensitivity analysis excluding ‘neutral’ responses from the ‘other’ (non-support) category was performed – data shown in Additional file 3. Respondents with healthier dietary habits (i.e., lower consumption of SSBs and higher consumption of FV) were also found to be more likely to support the three initiatives assessed – data shown in Additional file 4. Stratified analyses according to country showed that these associations differed in several respects (magnitude and statistical significance) between countries and initiatives – data shown in Additional file 5.

**Table 2:** Adjusted OR (95% CI) of characteristics associated with support for supermarket initiatives focused on product placement

Variable	Categories	Fewer end-of-aisle displays containing unhealthy foods or soft drinks		More shelf space for fresh and healthier foods		Checkouts with only healthy products	
		Support %	OR^a^ (95% CI)	Support %	OR^a^ (95% CI)	Support %	OR^a^ (95% CI)
Country	Australia	57	Reference	70	Reference	49	Reference
	Canada	54	**0.86 (0.75 to 0.97)**	69	0.92 (0.80 to 1.06)	44	**0.83 (0.73 to 0.94)**
	UK	58	**1.13 (1.00 to 1.28)**	67	0.95 (0.83 to 1.08)	49	1.10 (0.98 to 1.24)
	US	52	**0.80 (0.71 to 0.92)**	69	0.95 (0.82 to 1.09)	40	**0.70 (0.62 to 0.80)**
	Mexico	59	**1.19 (1.04 to 1.36)**	88	**3.58 (2.99 to 4.29)**	62	**1.77 (1.54 to 2.03)**
	Canada	54	Reference	69	Reference	44	Reference
	Australia	57	**1.17 (1.03 to 1.33)**	70	1.09 (0.95 to 1.26)	49	**1.20 (1.06 to 1.37)**
	UK	58	**1.33 (1.17 to 1.50)**	67	1.03 (0.90 to 1.18)	49	**1.33 (1.17 to 1.50)**
	US	52	0.94 (0.83 to 1.08)	69	1.03 (0.89 to 1.20)	40	**0.84 (0.74 to 0.96)**
	Mexico	59	**1.39 (1.21 to 1.60)**	88	**3.91 (3.25 to 4.69)**	62	**2.13 (1.85 to 2.46)**
	UK	58	Reference	67	Reference	49	Reference
	Canada	54	**0.75 (0.67 to 0.86)**	69	0.97 (0.84 to 1.11)	44	**0.75 (0.67 to 0.85)**
	Australia	57	0.88 (0.78 to 1.00)	70	1.06 (0.92 to 1.21)	49	0.91 (0.81 to 1.02)
	US	52	**0.71 (0.63 to 0.80)**	69	1.00 (0.87 to 1.15)	40	**0.64 (0.56 to 0.72)**
	Mexico	59	1.05 (0.92 to 1.20)	88	**3.78 (3.18 to 4.50)**	62	**1.61 (1.41 to 1.83)**
	US	52	Reference	69	Reference	40	Reference
	Canada	54	1.06 (0.93 to 1.21)	69	0.97 (0.84 to 1.12)	44	**1.18 (1.04 to 1.35)**
	Australia	57	**1.24 (1.09 to 1.41)**	70	1.06 (0.92 to 1.22)	49	**1.43 (1.26 to 1.62)**
	UK	58	**1.41 (1.24 to 1.59)**	67	1.00 (0.87 to 1.15)	49	**1.57 (1.39 to 1.78)**
	Mexico	59	**1.47 (1.29 to 1.69)**	88	**3.78 (3.17 to 4.51)**	62	**2.53 (2.20 to 2.89)**
	Mexico	59	Reference	88	Reference	62	Reference
	Canada	54	**0.72 (0.63 to 0.83)**	69	**0.26 (0.21 to 0.31)**	44	**0.47 (0.41 to 0.54)**
	Australia	57	**0.84 (0.73 to 0.97)**	70	**0.28 (0.23 to 0.33)**	49	**0.56 (0.49 to 0.65)**
	UK	58	0.95 (0.84 to 1.09)	67	**0.26 (0.22 to 0.31)**	49	**0.62 (0.55 to 0.71)**
	US	52	**0.68 (0.59 to 0.78)**	69	**0.26 (0.22 to 0.32)**	40	**0.40 (0.35 to 0.45)**
Age	18–29	49	Reference	68	Reference	44	Reference
	30–44	53	1.12 (0.99 to 1.25)	70	1.13 (0.99 to 1.29)	51	**1.27 (1.13 to 1.43)**
	45–59	58	**1.41 (1.25 to 1.59)**	75	**1.60 (1.38 to 1.84)**	51	**1.31 (1.16 to 1.48)**
	60+	64	**1.85 (1.64 to 2.10)**	76	**1.87 (1.63 to 2.16)**	48	**1.28 (1.13 to 1.44)**
Sex	Male	52	Reference	67	Reference	46	Reference
	Female	60	**1.41 (1.30 to 1.53)**	78	**1.74 (1.59 to 1.91)**	52	**1.31 (1.21 to 1.42)**
Education	Low	53	Reference	69	Reference	45	Reference
	Medium	58	**1.19 (1.07 to 1.32)**	71	**1.12 (1.00 to 1.26)**	50	**1.15 (1.04 to 1.28)**
	High	58	**1.19 (1.08 to 1.31)**	78	**1.25 (1.12 to 1.39)**	53	**1.15 (1.04 to 1.26)**
Nutrition knowledge	None/low	51	Reference	67	Reference	44	Reference
	Moderate	57	**1.23 (1.12 to 1.35)**	74	**1.26 (1.14 to 1.39)**	49	**1.17 (1.07 to 1.28)**
	High	63	**1.68 (1.50 to 1.88)**	81	**2.12 (1.84 to 2.43)**	59	**1.92 (1.71 to 2.15)**
BMI	< 18.5	57	Reference	75	Reference	50	Reference
	18.5–24.9	51	0.85 (0.66 to 1.10)	73	0.98 (0.72 to 1.34)	45	0.88 (0.68 to 1.14)
	25–30	58	1.01 (0.91 to 1.12)	74	0.96 (0.85 to 1.08)	50	1.04 (0.94 to 1.15)
	≥30	60	**1.14 (1.01 to 1.27)**	72	0.90 (0.79 to 1.03)	50	1.09 (0.97 to 1.22)
	Missing/not stated	45	**0.64 (0.56 to 0.73)**	62	**0.60 (0.52 to 0.70)**	43	**0.78 (0.68 to 0.89)**

^a^Adjusted for all other variables listed

Estimates of support (%) across categories are weighted

In bold: Statistically significant associations (*p* < 0.05)

Statistically significant interactions (*p* < 0.05) were observed between country and both age and self-reported nutrition knowledge in relation to support for ‘fewer end-of-aisle displays containing unhealthy foods or soft drinks’ (i.e., associations between these covariates and support for ‘fewer end-of-aisle displays containing unhealthy foods or soft drinks’ differed according to country). In the case of support for ‘checkouts with only healthy products’, significant interactions were observed between country and both age and sex. Finally, concerning support for ‘more shelf space for fresh and healthier foods’, there was a statistically significant interaction between country and self-reported nutrition knowledge.

## Discussion

This study assessed the level and determinants of public support for product placement interventions to encourage healthy food choices in supermarkets, with a focus on differences between and within countries. Most respondents in five countries supported the initiatives presented to them, although the level of support differed according to country and type of initiative (i.e., product positioning or availability). In line with previous evidence, initiatives that most limited freedom of choice, such as checkouts with only healthy products, generally had relatively lower support [30–36].

To our knowledge, this is the first study to explore sociodemographic differences, as well as differences between diverse countries, in relation to support for different types of product placement interventions in supermarkets. Sociodemographic characteristics of individuals, including age, sex, education, self-reported nutrition knowledge and dietary habits were found to be associated with level of support. Both the magnitude and statistical significance of these associations varied according to country, suggesting that country-level factors play an important role in public support for the assessed supermarket interventions.

### Differences between initiatives

Public support was lower for more restrictive measures related to product positioning (i.e., banning or limiting unhealthy foods from dynamic promotional displays). On the other hand, the most supported initiative overall and across countries targeted availability (i.e., more shelf space for fresh and healthier foods). The same trend of support was observed among Australian respondents (*n* = 3767) in the 2017 IFPS wave (surveys in the other four countries did not include the supermarket initiative questions in 2017). In the 2017 survey wave, the highest support was also reported for more shelf space for fresh and healthier foods, with lower support for imposing restrictions on placement of unhealthy products at highly visible locations within the store, including checkouts, end-of-aisle, and island bin displays (data not shown).

Maintaining a perception of choice has been identified as being core to promoting a sense of fairness, which, together with perceived effectiveness, are among the main predictors of policy acceptability [41, 42]. Therefore, the observed trends of higher support for initiatives targeting availability compared to support for product positioning initiatives could be a function of respondents feeling that the choice-preserving nature of nudges are compromised in product positioning interventions. Interestingly, a study that tested acceptability towards a similar nudge involving repositioning of food products (i.e., placing healthy foods at the cash register desk, while keeping unhealthy products available elsewhere in the shop) found that the majority of customers reported positive attitudes towards it [52]. This may suggest that framing more restrictive interventions in terms of both product positioning and availability could increase support for the least popular initiative in the present study (i.e. ‘checkouts with only healthy products’), as it was not explicitly specified that unhealthy foods would still be available elsewhere in the store. The impact of intervention framing on public support warrants further investigation.

In agreement with the current findings, it has also been reported that acceptability varies based on intervention-specific beliefs and the targeted food type, with more support observed for healthy food-related interventions [32, 33]. Existing evidence suggests that, although strategies that discourage unhealthy high-calorie choices are actually more effective than strategies that encourage low-calorie choices, the public tends to perceive interventions that encourage low-calorie choices to be more effective, fairer, and more acceptable than those that discourage high-calorie choices [33]. Therefore, it appears that a nudge is not approved or disapproved as such, it receives approval if and to the extent that people approve of the direction in which it nudges.

### Differences between countries

Support for all initiatives was generally lowest in the US and highest in Mexico, although between-country differences varied across initiatives. This pattern of results aligns with findings from previous studies showing that policy support is influenced by country-level individualist or collectivist beliefs [30]. In addition, support for healthy food interventions has been found to be related to the degree to which people attribute obesity to external factors like excessive availability of unhealthy foods [29, 32]. The individualistic perspective that is strongly institutionalized in Western countries [53], often irrespective of government political orientation [54], could also play a role in explaining the lower level of support for environmental interventions in Mexico compared to the US. These societal and attitudinal factors have been previously identified as having more explanatory power in terms of policy support than sociodemographic characteristics and political preferences [29].

Moreover, the culture in which an individual develops will condition the acceptable and desirable norms of behavior, so the highest support in Mexico may also reflect that Mexicans have higher levels of acquiescent responses or social desirability (i.e., tendency of survey respondents to answer questions in a manner that will be viewed favorably by others). Consistent with this perspective, previous research has found that acquiescence differs across Latino ethnic subgroups in the US [55] and that Mexicans generally demonstrate a strong desire to please others and seek approval [56].

Support could also be expected to be more favorable among countries where these kind of interventions have already been adopted, as it has been shown that policies tend to become more acceptable after they have been implemented [32]. Interestingly, support for healthy checkouts was highest in Mexico despite retailers in the UK making significant progress in this area [16]. This finding may also be related to an increased awareness of the need for action to tackle the current epidemic of obesity and diabetes in Mexico [57]. Furthermore, while Mexico is undergoing a radical transformation in its food system and diet away from its indigenous roots, public markets, street markets and informal retailers continue to be principal actors in urban food provisioning despite the incursion of corporate retailers [58]. Nevertheless, it is noteworthy that the Mexico sample had notably higher levels of education than census estimates, so the distortion of observed estimates due to residual confounding by high education cannot be ruled out.

### Differences within countries

Overall, respondents who were older, female, highly educated, and reported having more nutrition knowledge tended to be more supportive of the supermarket initiatives.

Several explanations have been proposed for the greater support observed among certain demographic sub-groups, such as increased health consciousness among women and enhanced awareness of the burden of disease with age [32]. Similarly, an individual’s awareness and experience of harm associated with the target behavior also seems to influence support [32]. For instance, knowledge of harm has been found to increase support for policies designed to restrict smoking and second-hand smoke. In this study, there was a statistically significant direct association between nutrition knowledge and support for healthy supermarket-led initiatives. Exploratory analyses (not shown) suggested that those participants aware of the link between consumption of sugary drinks/ food high in sugar, and increased risk of chronic health conditions like obesity and diabetes, were significantly more likely to support all initiatives. Respondents’ own behavior, health and experience have also been consistently associated with support, being highest among those whose habits are not discouraged [32]. Although self-reported consumption of SSB and FV did not substantially alter the results when included as covariates in logistic regression models, participants with less healthy dietary habits (i.e., high SSB and low FV consumption) were found to be more likely to reject interventions that aim to discourage these habits. This finding is consistent with previous evidence showing that self-interest is an important predictor of individual preferences for interventions [32].

Additionally, having a higher BMI was marginally associated with support for fewer end-of-aisle displays containing unhealthy foods or soft drinks but not with support for other initiatives. However, a significant proportion of participants in each country did not report their BMI or were coded as missing after deleting extreme numeric values, with this group having the lowest support for all initiatives. These findings seem to contradict each other given that this set of participants often have larger body sizes [59]. Evidence regarding BMI and support for health promoting policies has also been inconsistent, with one previous study finding that those with a higher BMI were more likely to support menu labelling policies [60], and another that those with a higher BMI had less positive attitudes towards the use of food labels [61].

Statistically significant interactions were found between country and several covariates including age, sex, and self-reported nutrition knowledge, with no common trend across initiatives. This shows that country-level culture and ideology play an important role on the magnitude and direction of the association between sociodemographic characteristics and support for supermarket placement initiatives. Further studies would be required to better understand the interactions between context and individual-level factors.

### Strengths and limitations

The main strengths of this study include the comparison of multiple countries based on a relatively large sample size. To our knowledge, this is the most comprehensive multi-country study exploring support for different initiatives to promote healthy food choices in supermarkets and considering a range of sociodemographic variables. It is also worth highlighting that this is the first study to assess supermarket nudges in low or middle-income countries in comparison with higher income countries. Considering that previous studies have been mainly carried out in Europe and the United States, the present study adds to the existing knowledge and sheds light on the public health relevance of these kind of initiatives in Latin America. However, some limitations should be acknowledged. Firstly, we only assessed support for three initiatives focused on product placement. Given the broad scope of the IFPS, which assesses many different domains related to dietary patterns and policy-relevant behaviors, it was only possible to include a very limited number of questions regarding support for supermarket policies within the retail environment section of the survey. A broader set of different types of interventions (e.g., based on education, price, food labelling, etc.) should be explored in future studies in order to better understand which types of retailer interventions are likely to attract more support. Also, this study did not assess whether support varied depending on whether the intervention is led by supermarkets or regulated by governments; this area warrants further investigation. Although sampling weights were applied, the sample cannot be considered to be nationally representative as respondents were recruited using non-probability-based sampling. For example, the study sample differed from the general population across the five countries with a somewhat lower proportion of self-reported overweight and obese individuals compared to national estimates. Moreover, as mentioned earlier, the Mexican recruitment panel had few available subjects with low education so the study sample is biased toward participation of more highly educated individuals from Mexico. All self-reported data are subject to inherent measurement error. Nevertheless, all were derived from previously published instruments. Lastly, given the snapshot nature of cross-sectional studies, we cannot establish causality for any association. Even though this study design is appropriate for determining the current level of support for supermarket interventions among demographic sub-groups and how it varies between countries, comparisons using repeated measures from future waves of the IFPS study will be insightful to evaluate changes over time, particularly in light of any country-specific changes in supermarket layouts or policies.

### Implications

This large, multi-country study represents a unique opportunity to assess the support for different healthy food retail initiatives in the supermarket setting. Although we were only able to assess a limited range of initiatives in this survey, the results confirm that the majority of respondents supported supermarket initiatives focused on product placement and, in particular, greater shelf space for fruits and vegetables. In most contexts, there could be an opportunity to further increase support by targeting customers’ nutrition knowledge. This evidence can be used by governments, public health groups and civil society organizations to advocate for healthier supermarket food environments, and should also encourage supermarkets to innovate in this regard. Local consultation with retailers may be helpful to understand the degree to which these findings could feasibly influence their marketing practices.

## Conclusions

Although support varied somewhat based on the type of initiative, the cultural context of countries and individual sociodemographic characteristics, most people in the five included countries supported supermarket initiatives focused on product placement. This evidence should be used to support efforts by retailers and policy makers to implement healthy food retail strategies including healthier checkouts and end-of-aisle displays, as well as increased shelf space for fruit and vegetables.

## Supplementary Information

**Additional file 1: Supplementary Table 1.** Characteristics of the overall analytic sample vs. those with missing values (not asked) in each supermarket initiative support question. International Food Policy Study 2018 (*n* = 22,264). Unweighted.

**Additional file 2: Supplementary Table 2.** Weighted proportion (%) of ‘support’ (S), ‘neutral’ (N) and ‘oppose’ (O) responses to supermarket initiatives in the total sample and by country. International Food Policy Study 2018 (*n* = 22,264).

**Additional file 3: Supplementary Table 3.** Adjusted OR* (95% CI) of characteristics associated with support for supermarket initiatives focused on product placement – excluding respondents that selected ‘neutral’. International Food Policy Study 2018.

**Additional file 4: Supplementary Table 4.** Adjusted OR* (95% CI) of characteristics associated with support for supermarket initiatives focused on product placement – including dietary variables. International Food Policy Study 2018.

**Additional file 5: Supplementary Table 5.** Adjusted OR* (95% CI) of characteristics associated with support for supermarket initiatives stratified by countries. International Food Policy Study 2018.

## Data Availability

The datasets used and/or analyzed during the current study are available from the corresponding author on reasonable request.

## Abbreviations

BMI: Body Mass Index
FV: Fruits and Vegetables
IFPS: International Food Policy Study
UK: United Kingdom
US: United States
