# Supplementary material for: Public support for healthy supermarket initiatives focused on product placement: a multi-country cross-sectional analysis of the 2018 International Food Policy Study
Source: Int J Behav Nutr Phys Act. 2021 Jun 14;18:78. doi: 10.1186/s12966-021-01149-0 (PMC8201822; doi:10.1186/s12966-021-01149-0)
Supplement: Supplementary file 1 — Additional file 1: Supplementary Table 1. Characteristics of the overall analytic sample vs. those with missing values (not asked) in each supermarket initiative support question. International Food Policy Study 2018 (n = 22,264). Unweighted. [file 12966_2021_1149_MOESM1_ESM.pdf]

Supplementary Table 1. Characteristics of the overall analytic sample vs. those with missing values (not asked) in each supermarket initiative support question. International Food Policy Study 2018 (n=22,264). Unweighted.

	**Overall** *(n=22,264)*	**End-of-aisle** *(n=7,398)*	**Shelf space** *(n=7,427)*	**Checkouts** *(n=7,439)*
**Country**				
Australia	4,004 (18.0)	1,340 (19.1)	1,326 (17.9)	1,338 (18.0)
Canada	4,288 (19.3)	1,416 (18.1)	1,402 (18.9)	1,470 (19.7)
United Kingdom	5,367 (24.1)	1,753 (23.7)	1,811 (24.4)	1,803 (24.2)
United States	4,523 (20.3)	1,511 (20.4)	1,519 (20.4)	1,493 (20.1)
Mexico	4,082 (18.3)	1,378 (18.6)	1,369 (18.4)	1,335 (18.0)
**Sex**				
Male	10,938 (49.1)	3,676 (49.7)	3,661 (49.3)	3,601 (48.4)
Female	11,326 (50.9)	3,722 (50.3)	3,766 (50.7)	3,838 (51.6)
**Age group**				
18-29 years old	4,693 (21.1)	1,585 (21.4)	1,548 (20.8)	1,560 (21.0)
30-44 years old	6,046 (27.2)	2,046 (27.7)	2,017 (27.2)	1,983 (26.7)
45-59 years old	5,258 (23.6)	1,711 (23.1)	1,825 (24.6)	1,722 (23.1)
60+ years old	6,267 (28.1)	2,056 (27.8)	2,037 (27.4)	2,174 (29.2)
**Ethnicity**				
Majority	18,368 (82.5)	6,089 (82.3)	6,125 (82.5)	6,154 (82.7)
Minority	3,636 (16.3)	1,220 (16.5)	1,216 (16.4)	1,200 (16.1)
Not stated	260 (1.2)	89 (1.2)	86 (1.2)	85 (1.2)
**Education**				
Low	5,980 (26.8)	1,972 (26.6)	1,995 (26.9)	2,013 (27.1)
Medium	6,051 (27.2)	2,012 (27.2)	1,993 (26.8)	2,046 (27.5)
High	10,174 (45.7)	3,400 (46.0)	3,416 (46.0)	3,358 (45.1)
Not stated	59 (0.3)	14 (0.2)	23 (0.3)	22 (0.3)
**BMI**				
<18.5	616 (2.8)	208 (2.8)	219 (3.0)	189 (2.5)
18.5-24.9	7,921 (35.6)	2,621 (35.4)	2,580 (34.7)	2,720 (36.6)
25-30	6,411 (28.8)	2,138 (28.9)	2,163 (29.1)	2,110 (28.4)
≥30	4,550 (20.4)	1,516 (20.5)	1,539 (20.7)	1,495 (20.1)
Missing/not stated	2,766 (12.4)	915 (12.4)	926 (12.5)	925 (12.4)
**Nutrition knowledge**				
None/low	7,960 (35.7)	2,639 (35.7)	2,698 (36.3)	2,623 (35.2)
Moderate	9,591 (43.1)	3,229 (43.7)	3,142 (42.3)	3,220 (43.3)
High	4,626 (20.8)	1,500 (20.3)	1,557 (21.0)	1,569 (21.1)
Not stated	87 (0.4)	30 (0.4)	30 (0.4)	27 (0.4)
