# Supplementary material for: Public support for healthy supermarket initiatives focused on product placement: a multi-country cross-sectional analysis of the 2018 International Food Policy Study
Source: Int J Behav Nutr Phys Act. 2021 Jun 14;18:78. doi: 10.1186/s12966-021-01149-0 (PMC8201822; doi:10.1186/s12966-021-01149-0)
Supplement: Supplementary file 2 — Additional file 2: Supplementary Table 2. Weighted proportion (%) of ‘support’ (S), ‘neutral’ (N) and ‘oppose’ (O) responses to supermarket initiatives in the total sample and by country. International Food Policy Study 2018 (n = 22,264). [file 12966_2021_1149_MOESM2_ESM.pdf]

Supplementary Table 2. Weighted proportion (%) of 'support' (S), 'neutral' (N) and 'oppose' (O) responses to supermarket initiatives in the total sample and by country. International Food Policy Study 2018 (n=22,264).

	**Overall (n=22,264)**			
	Respondents (n=14,866)			***Not asked (n=7,398)***
	**S**	**N**	**O**	
**Fewer end-of-aisle displays containing unhealthy foods or soft drinks**	55.9	36.7	7.4	–
	Respondents (n=14,825)			***Not asked (n=7,439)***
**Checkouts with only healthy products**	48.6	37.2	14.2	–
	Respondents (n=14,837)			***Not asked (n=7,427)***
**More shelf space for fresh and healthier foods**	72.0	25.5	2.5	–
	**Australia (n=4,004)**			
	Respondents (n=2,664)			***Not asked (n=1,340)***
**Fewer end-of-aisle displays containing unhealthy foods or soft drinks**	56.8	35.8	7.5	–
	Respondents (n=2,666)			***Not asked (n=1,338)***
**Checkouts with only healthy products**	48.5	37.8	13.7	–
	Respondents (n=2,678)			***Not asked (n=1,326)***
**More shelf space for fresh and healthier foods**	69.5	27.9	2.6	–
	**Canada (n=4,288)**			
	Respondents (n= 2,872)			***Not asked (n=1,416)***
**Fewer end-of-aisle displays containing unhealthy foods or soft drinks**	53.6	39.3	7.1	–
	Respondents (n= 2,818)			***Not asked (n=1,470)***
**Checkouts with only healthy products**	44.3	39.8	15.9	–
	Respondents (n= 2,886)			***Not asked (n= 1,402)***
**More shelf space for fresh and healthier foods**	68.5	29.4	2.1	–
	**United Kingdom (n=5,367)**			
	Respondents (n=3,614)			***Not asked (n=1,753)***
**Fewer end-of-aisle displays containing unhealthy foods or soft drinks**	58.0	35.4	6.6	–
	Respondents (n=3,564)			***Not asked (n=1,803)***
**Checkouts with only healthy products**	49.3	36.0	14.7	–
	Respondents (n=3,556)			***Not asked (n=1,811)***
**More shelf space for fresh and healthier foods**	67.1	29.8	3.1	–
	**United States (n=4,523)**			
	Respondents (n=3,012)			***Not asked (n=1,511)***
**Fewer end-of-aisle displays containing unhealthy foods or soft drinks**	51.7	38.7	9.6	–
	Respondents (n=3,030)			***Not asked (n=1,493)***
**Checkouts with only healthy products**	40.1	39.4	20.5	–
	Respondents (n=3,004)			***Not asked (n=1,519)***
**More shelf space for fresh and healthier foods**	68.8	27.7	3.5	–
	**Mexico (n=4,082)**			
	Respondents (n=2,704)			***Not asked (n=1,378)***
**Fewer end-of-aisle displays containing unhealthy foods or soft drinks**	59.4	34.0	6.6	–
	Respondents (n=2,747)			***Not asked (n= 1,335)***
**Checkouts with only healthy products**	61.5	33.0	5.5	–
	Respondents (n=2,713)			***Not asked (n=1,369)***
**More shelf space for fresh and healthier foods**	88.3	10.7	1.0	–
