# Supplementary material for: Public support for healthy supermarket initiatives focused on product placement: a multi-country cross-sectional analysis of the 2018 International Food Policy Study
Source: Int J Behav Nutr Phys Act. 2021 Jun 14;18:78. doi: 10.1186/s12966-021-01149-0 (PMC8201822; doi:10.1186/s12966-021-01149-0)
Supplement: Supplementary file 3 — Additional file 3: Supplementary Table 3. Adjusted OR* (95% CI) of characteristics associated with support for supermarket initiatives focused on product placement – excluding respondents that selected ‘neutral’. International Food Policy Study 2018. [file 12966_2021_1149_MOESM3_ESM.pdf]

Supplementary Table 3. Adjusted OR* (95% CI) of characteristics associated with support for supermarket initiatives focused on product placement excluding respondents that selected 'neutral'. International Food Policy Study2018.

Variable	Categories	OR* (95% CI)		
		**Fewer end-of-aisle displays containing unhealthy foods or soft drinks**	**More shelf space for fresh and healthier foods**	**Checkouts with only healthy products**
**Country**	Australia	Reference	Reference	Reference
	Canada	0.92 (0.70 to 1.22)	1.21 (0.81 to 1.81)	**0.76 (0.62 to 0.93)**
	UK	**1.29 (1.00 to 1.67)**	0.89 (0.62 to 1.27)	1.01 (0.84 to 1.22)
	US	**0.72 (0.56 to 0.93)**	0.71 (0.49 to 1.03)	**0.54 (0.44 to 0.65)**
	Mexico	1.17 (0.88 to 1.55)	**3.48 (2.10 to 5.76)**	**3.05 (2.33 to 4.00)**
	Canada	Reference	Reference	Reference
	Australia	1.08 (0.82 to 1.43)	0.83 (0.55 to 1.23)	**1.31 (1.08 to 1.60)**
	UK	**1.40 (1.06 to 1.85)**	0.73 (0.49 to 1.09)	**1.33 (1.09 to 1.60)**
	US	0.78 (0.60 to 1.02)	0.58 (0.39 to 0.88)	**0.70 (0.58 to 0.85)**
	Mexico	1.26 (0.94 to 1.69)	**2.87 (1.70 to 4.85)**	**4.01 (3.06 to 5.26)**
	UK	Reference	Reference	Reference
	Canada	**0.71 (0.54 to 0.94)**	1.37 (0.92 to 2.03)	**0.75 (0.62 to 0.91)**
	Australia	0.77 (0.60 to 1.00)	1.13 (0.79 to 1.61)	0.99 (0.82 to 1.20)
	US	**0.56 (0.44 to 0.71)**	0.80 (0.55 to 1.16)	**0.53 (0.45 to 0.63)**
	Mexico	0.90 (0.69 to 1.18)	**3.92 (2.42 to 6.35)**	**3.03 (2.33 to 3.92)**
	US	Reference	Reference	Reference
	Canada	1.28 (0.98 to 1.67)	**1.72 (1.14 to 2.59)**	**1.42 (1.17 to 1.72)**
	Australia	**1.39 (1.08 to 1.78)**	1.42 (0.98 to 2.06)	**1.87 (1.55 to 2.25)**
	UK	**1.79 (1.41 to 2.29)**	1.26 (0.86 to 1.82)	**1.88 (1.58 to 2.24)**
	Mexico	**1.62 (1.25 to 2.09)**	**4.93 (3.01 to 8.06)**	**5.70 (4.41 to 7.36)**
	Mexico	Reference	Reference	Reference
	Canada	0.79 (0.59 to 1.06)	**0.35 (0.21 to 0.59)**	**0.25 (0.19 to 0.33)**
	Australia	0.86 (0.65 to 1.14)	**0.29 (0.17 to 0.48)**	**0.33 (0.25 to 0.43)**
	UK	1.11 (0.85 to 1.46)	**0.25 (0.16 to 0.41)**	**0.33 (0.25 to 0.43)**
	US	**0.62 (0.48 to 0.80)**	**0.20 (0.12 to 0.33)**	**0.18 (0.14 to 0.23)**
**Age**	18-29	Reference	Reference	Reference
	30-44	1.11 (0.88 to 1.40)	1.09 (0.77 to 1.54)	**1.36 (1.11 to 1.66)**
	45-59	**1.35 (1.05 to 1.72)**	**1.62 (1.09 to 2.41)**	1.03 (0.85 to 1.26)
	60+	**1.63 (1.28 to 2.09)**	**1.81 (1.21 to 2.71)**	0.88 (0.73 to 1.07)
**Sex**	Male	Reference	Reference	Reference
	Female	**1.81 (1.53 to 2.14)**	**3.38 (2.57 to 4.44)**	**1.60 (1.41 to 1.82)**
**Education**	Low	Reference	Reference	Reference
	Medium	**1.40 (1.13 to 1.73)**	0.90 (0.66 to 1.23)	**1.31 (1.12 to 1.53)**
	High	**1.51 (1.26 to 1.81)**	1.15 (0.86 to 1.54)	**1.30 (1.13 to 1.50)**
**Nutrition knowledge**	None/low	Reference	Reference	Reference
	Moderate	**1.22 (1.02 to 1.47)**	**1.55 (1.15 to 2.07)**	**1.19 (1.04 to 1.37)**
	High	**1.37 (1.08 to 1.73)**	**1.60 (1.11 to 2.30)**	**2.07 (1.72 to 2.48)**
**BMI**	<18.5	Reference	Reference	Reference
	18.5-24.9	**0.58 (0.37 to 0.92)**	0.47 (0.24 to 0.92)	1.28 (0.85 to 1.94)
	25-30	0.99 (0.80 to 1.22)	0.83 (0.59 to 1.15)	1.06 (0.90 to 1.24)
	≥30	1.12 (0.88 to 1.42)	0.90 (0.61 to 1.32)	1.06 (0.89 to 1.26)
	Missing/not stated	**0.59 (0.45 to 0.77)**	**0.44 (0.30 to 0.64)**	**0.80 (0.64 to 1.00)**

*Adjusted for all other variables listed.
Estimates of support (%) across categories are weighted.
In bold: Statistically significant associations (p<0.05).
