# Supplementary material for: Public support for healthy supermarket initiatives focused on product placement: a multi-country cross-sectional analysis of the 2018 International Food Policy Study
Source: Int J Behav Nutr Phys Act. 2021 Jun 14;18:78. doi: 10.1186/s12966-021-01149-0 (PMC8201822; doi:10.1186/s12966-021-01149-0)
Supplement: Supplementary file 4 — Additional file 4: Supplementary Table 4. Adjusted OR* (95% CI) of characteristics associated with support for supermarket initiatives focused on product placement – including dietary variables. International Food Policy Study 2018. [file 12966_2021_1149_MOESM4_ESM.pdf]

Supplementary Table 4. Adjusted OR* (95% CI) of characteristics associated with support for supermarket initiatives focused on product placement – including dietary variables. International Food Policy Study 2018.

Variable	Categories	Fewer end-of-aisle displays containing unhealthy foods or soft drinks	More shelf space for fresh and healthier foods	Checkouts with only healthy products
**Country**	US	Reference	Reference	Reference
	Canada	1.03 (0.89 to 1.19)	0.88 (0.74 to 1.05)	1.10 (0.95 to 1.28)
	Australia	**1.27 (1.09 to 1.47)**	1.02 (0.86 to 1.22)	**1.46 (1.26 to 1.69)**
	UK	**1.42 (1.23 to 1.64)**	0.91 (0.77 to 1.07)	**1.59 (1.38 to 1.83)**
	Mexico	**1.43 (1.22 to 1.67)**	**3.52 (2.84 to 4.35)**	**2.44 (2.08 to 2.86)**
**Age**	18-29	Reference	Reference	Reference
	30-44	1.12 (0.98 to 1.28)	1.14 (0.97 to 1.34)	**1.27 (1.10 to 1.45)**
	45-59	**1.34 (1.16 to 1.55)**	**1.47 (1.23 to 1.75)**	**1.20 (1.04 to 1.38)**
	60+	**1.66 (1.43 to 1.92)**	**1.46 (1.23 to 1.73)**	1.12 (0.97 to 1.30)
**Sex**	Male	Reference	Reference	Reference
	Female	**1.34 (1.22 to 1.47)**	**1.76 (1.57 to 1.97)**	**1.29 (1.18 to 1.42)**
**Education**	Low	Reference	Reference	Reference
	Medium	1.12 (0.99 to 1.27)	1.09 (0.95 to 1.26)	**1.13 (1.00 to 1.27)**
	High	**1.14 (1.02 to 1.27)**	**1.15 (1.01 to 1.31)**	1.07 (0.96 to 1.20)
**BMI**	<18.5	Reference	Reference	Reference
	18.5-24.9	0.80 (0.61 to 1.06)	0.96 (0.68 to 1.36)	0.93 (0.70 to 1.25)
	25-30	1.01 (0.90 to 1.14)	1.02 (0.88 to 1.17)	1.05 (0.93 to 1.17)
	≥30	**1.18 (1.03 to 1.34)**	0.97 (0.83 to 1.13)	**1.14 (1.00 to 1.29)**
	Missing/not stated	**0.70 (0.59 to 0.84)**	**0.67 (0.55 to 0.82)**	0.95 (0.80 to 1.13)
**Nutrition knowledge**	None/low	Reference	Reference	Reference
	Moderate	**1.19 (1.07 to 1.32)**	**1.23 (1.08 to 1.39)**	**1.14 (1.03 to 1.27)**
	High	**1.52 (1.33 to 1.74)**	**1.85 (1.57 to 2.19)**	**1.69 (1.48 to 1.93)**
**SSB weekly consumption**	High	Reference	Reference	Reference
	Low	**1.20 (1.06 to 1.35)**	1.04 (0.90 to 1.19)	1.09 (0.97 to 1.23)
	None	**1.28 (1.13 to 1.44)**	**1.17 (1.01 to 1.35)**	**1.28 (1.14 to 1.45)**
**F&V daily consumption**	Low (<3 serv)	Reference	Reference	Reference
	Moderate (3-5)	1.10 (0.99 to 1.23)	**1.52 (1.33 to 1.73)**	**1.16 (1.04 to 1.30)**
	High (≥5 serv)	**1.16 (1.03 to 1.31)**	**1.64 (1.42 to 1.89)**	**1.28 (1.14 to 1.43)**

*Adjusted for all variables listed.

In bold: Statistically significant associations ($p<0.05$).
