# Supplementary material for: Public support for healthy supermarket initiatives focused on product placement: a multi-country cross-sectional analysis of the 2018 International Food Policy Study
Source: Int J Behav Nutr Phys Act. 2021 Jun 14;18:78. doi: 10.1186/s12966-021-01149-0 (PMC8201822; doi:10.1186/s12966-021-01149-0)
Supplement: Supplementary file 5 — Additional file 5: Supplementary Table 5. Adjusted OR* (95% CI) of characteristics associated with support for supermarket initiatives stratified by countries. International Food Policy Study 2018. [file 12966_2021_1149_MOESM5_ESM.pdf]

Supplementary Table 5. Adjusted OR* (95% CI) of characteristics associated with support for supermarket initiatives stratified by countries. International Food Policy Study 2018.

**Country**	**Supermarket initiatives**		
**Australia**	**Fewer end-of-aisle displays containing unhealthy foods or soft drinks**	**More shelf space for fresh and healthier foods**	**Checkouts with only healthy products**
**Age**			
18-29	Reference	Reference	Reference
30-44	0.97 (0.73 to 1.28)	1.33 (0.99 to 1.79)	1.06 (0.80 to 1.41)
45-59	**1.87 (1.40 to 2.50)**	**2.07 (1.52 to 2.83)**	**1.97 (1.48 to 2.63)**
60+	**2.20 (1.66 to 2.92)**	**2.11 (1.56 to 2.86)**	**1.47 (1.12 to 1.93)**
**Sex**			
Male	Reference	Reference	Reference
Female	**1.54 (1.28 to 1.86)**	**1.72 (1.40 to 2.11)**	**1.38 (1.15 to 1.66)**
**Education**			
Low	Reference	Reference	Reference
Medium	1.17 (0.94 to 1.47)	1.11 (0.88 to 1.42)	**1.25 (1.01 to 1.55)**
High	1.24 (0.97 to 1.58)	1.11 (0.85 to 1.44)	1.20 (0.94 to 1.52)
**Nutrition knowledge**			
None/low	Reference	Reference	Reference
Moderate	1.17 (0.95 to 1.45)	1.20 (0.96 to 1.50)	1.14 (0.93 to 1.39)
High	**1.65 (1.28 to 2.13)**	**2.17 (1.61 to 2.92)**	**1.90 (1.49 to 2.43)**
**BMI**			
<18.5	Reference	Reference	Reference
18.5-24.9	0.86 (0.49 to 1.51)	0.63 (0.34 to 1.19)	0.68 (0.38 to 1.22)
25-30	1.02 (0.81 to 1.30)	0.94 (0.72 to 1.22)	0.90 (0.71 to 1.13)
≥30	1.19 (0.92 to 1.54)	1.06 (0.80 to 1.41)	0.97 (0.76 to 1.24)
Missing/not stated	**0.61 (0.45 to 0.82)**	**0.47 (0.34 to 0.64)**	**0.48 (0.36 to 0.66)**
**Canada**	**Fewer end-of-aisle displays containing unhealthy foods or soft drinks**	**More shelf space for fresh and healthier foods**	**Checkouts with only healthy products**
**Age**			
18-29	Reference	Reference	Reference
30-44	1.14 (0.85 to 1.53)	1.28 (0.94 to 1.75)	1.13 (0.84 to 1.53)
45-59	**1.47 (1.09 to 1.97)**	**1.66 (1.20 to 2.30)**	1.27 (0.94 to 1.71)
60+	**2.12 (1.58 to 2.86)**	**2.23 (1.60 to 3.09)**	1.31 (0.97 to 1.77)
**Sex**			
Male	Reference	Reference	Reference
Female	**1.38 (1.13 to 1.67)**	**1.86 (1.50 to 2.29)**	**1.32 (1.09 to 1.59)**
**Education**			
Low	Reference	Reference	Reference
Medium	**1.42 (1.13 to 1.78)**	1.19 (0.93 to 1.52)	**1.21 (0.97 to 1.51)**
High	**1.43 (1.13 to 1.81)**	1.22 (0.95 to 1.56)	1.16 (0.91 to 1.47)
**Nutrition knowledge**			
None/low	Reference	Reference	Reference
Moderate	**1.45 (1.16 to 1.80)**	**1.28 (1.01 to 1.62)**	1.12 (0.90 to 1.39)
High	**1.91 (1.49 to 2.46)**	**1.96 (1.47 to 2.61)**	**1.94 (1.51 to 2.50)**
**BMI**			
<18.5	Reference	Reference	Reference
18.5-24.9	0.67 (0.38 to 1.17)	1.36 (0.73 to 2.56)	0.76 (0.43 to 1.34)
25-30	0.87 (0.68 to 1.10)	**0.73 (0.56 to 0.96)**	0.97 (0.77 to 1.24)
≥30	1.24 (0.95 to 1.62)	**0.74 (0.55 to 0.98)**	1.19 (0.93 to 1.53)
Missing/not stated	**0.52 (0.38 to 0.73)**	**0.46 (0.33 to 0.66)**	**0.66 (0.46 to 0.95)**

Country	Supermarket initiatives		
**UK**	**Fewer end-of-aisle displays containing unhealthy foods or soft drinks**	**More shelf space for fresh and healthier foods**	**Checkouts with only healthy products**
**Age**			
18-29	Reference	Reference	Reference
30-44	**1.44 (1.12 to 1.86)**	1.04 (0.80 to 1.36)	**1.51 (1.17 to 1.95)**
45-59	**1.95 (1.50 to 2.53)**	**1.77 (1.33 to 2.34)**	**1.65 (1.27 to 2.14)**
60+	**2.68 (2.08 to 3.45)**	**1.97 (1.52 to 2.55)**	**1.77 (1.38 to 2.29)**
**Sex**			
Male	Reference	Reference	Reference
Female	**1.38 (1.16 to 1.63)**	**1.63 (1.37 to 1.95)**	**1.47 (1.25 to 1.73)**
**Education**			
Low	Reference	Reference	Reference
Medium	1.15 (0.93 to 1.41)	1.10 (0.89 to 1.38)	1.15 (0.94 to 1.41)
High	**1.20 (1.00 to 1.45)**	**1.26 (1.03 to 1.54)**	**1.30 (1.08 to 1.56)**
**Nutrition knowledge**			
None/low	Reference	Reference	Reference
Moderate	1.11 (0.92 to 1.33)	1.05 (0.87 to 1.27)	**1.19 (1.00 to 1.42)**
High	**1.41 (1.11 to 1.79)**	**1.82 (1.37 to 2.40)**	**1.89 (1.49 to 2.39)**
**BMI**			
<18.5	Reference	Reference	Reference
18.5-24.9	1.00 (0.62 to 1.60)	1.02 (0.59 to 1.75)	1.06 (0.65 to 1.74)
25-30	0.94 (0.76 to 1.16)	1.00 (0.79 to 1.26)	1.11 (0.90 to 1.36)
≥30	1.06 (0.82 to 1.37)	0.93 (0.71 to 1.21)	0.93 (0.73 to 1.19)
Missing/not stated	**0.73 (0.57 to 0.93)**	**0.73 (0.57 to 0.94)**	0.79 (0.62 to 1.00)
**US**	**Fewer end-of-aisle displays containing unhealthy foods or soft drinks**	**More shelf space for fresh and healthier foods**	**Checkouts with only healthy products**
**Age**			
18-29	Reference	Reference	Reference
30-44	0.77 (0.58 to 1.01)	1.02 (0.75 to 1.39)	**1.57 (1.20 to 2.05)**
45-59	0.83 (0.62 to 1.10)	1.21 (0.88 to 1.66)	0.95 (0.71 to 1.27)
60+	1.09 (0.83 to 1.42)	**1.50 (1.11 to 2.02)**	0.91 (0.69 to 1.18)
**Sex**			
Male	Reference	Reference	Reference
Female	**1.69 (1.41 to 2.03)**	**2.08 (1.69 to 2.56)**	**1.52 (1.26 to 1.84)**
**Education**			
Low	Reference	Reference	Reference
Medium	1.07 (0.87 to 1.33)	1.15 (0.91 to 1.46)	1.11 (0.88 to 1.39)
High	1.24 (1.03 to 1.50)	**1.50 (1.22 to 1.86)**	1.21 (0.99 to 1.46)
**Nutrition knowledge**			
None/low	Reference	Reference	Reference
Moderate	**1.58 (1.28 to 1.94)**	**1.59 (1.27 to 1.99)**	**1.29 (1.03 to 1.60)**
High	**2.17 (1.69 to 2.77)**	**2.86 (2.13 to 3.85)**	**2.31 (1.81 to 2.96)**
**BMI**			
<18.5	Reference	Reference	Reference
18.5-24.9	0.78 (0.46 to 1.34)	0.90 (0.42 to 1.95)	1.21 (0.70 to 2.08)
25-30	1.05 (0.84 to 1.33)	1.04 (0.80 to 1.35)	0.96 (0.76 to 1.22)
≥30	1.07 (0.84 to 1.37)	0.87 (0.66 to 1.14)	1.10 (0.86 to 1.41)
Missing/not stated	**0.64 (0.46 to 0.87)**	**0.65 (0.45 to 0.94)**	0.84 (0.61 to 1.16)
**Mexico**	**Fewer end-of-aisle displays containing unhealthy foods or soft drinks**	**More shelf space for fresh and healthier foods**	**Checkouts with only healthy products**

**Age**			
18-29	Reference	Reference	Reference
30-44	**1.32 (1.08 to 1.64)**	1.03 (0.76 to 1.40)	1.12 (0.91 to 1.40)
45-59	**1.32 (1.02 to 1.71)**	1.43 (0.95 to 2.15)	1.09 (0.84 to 1.41)
60+	1.40 (0.92 to 2.14)	1.37 (0.67 to 2.81)	1.02 (0.67 to 1.54)
**Sex**			
Male	Reference	Reference	Reference
Female	1.16 (0.96 to 1.40)	**1.35 (1.01 to 1.80)**	0.93 (0.76 to 1.13)
**Education**			
Low	Reference	Reference	Reference
Medium	1.19 (0.83 to 1.71)	0.75 (0.45 to 1.27)	1.10 (0.77 to 1.58)
High	1.07 (0.84 to 1.37)	1.02 (0.70 to 1.49)	1.06 (0.82 to 1.36)
**Nutrition knowledge**			
None/low	Reference	Reference	Reference
Moderate	1.00 (0.81 to 1.24)	1.34 (0.99 to 1.82)	1.11 (0.90 to 1.37)
High	1.32 (0.98 to 1.80)	**1.68 (1.05 to 2.69)**	1.30 (0.94 to 1.79)
**BMI**			
<18.5	Reference	Reference	Reference
18.5-24.9	1.20 (0.58 to 2.46)	1.67 (0.64 to 4.37)	0.58 (0.31 to 1.06)
25-30	1.14 (0.91 to 1.44)	1.21 (0.84 to 1.73)	1.19 (0.95 to 1.50)
≥30	1.08 (0.81 to 1.45)	1.01 (0.67 to 1.52)	1.21 (0.90 to 1.62)
Missing/not stated	**0.68 (0.50 to 0.93)**	0.70 (0.45 to 1.07)	1.30 (0.94 to 1.81)

*Adjusted for all variables listed.

In bold: Statistically significant associations (p<0.05).
